# Entropy Method for Decision Making with Uncertainty

**DOI:** 10.3390/e28020141

**Published:** 2026-01-27

**Authors:** Małgorzata Przybyła-Kasperek

**Affiliations:** Institute of Computer Science, University of Silesia in Katowice, Bedzinska 39, 41-200 Sosnowiec, Poland; malgorzata.przybyla-kasperek@us.edu.pl; Tel.: +48-32-269-17-56

## 1. Introduction

In complex socio-technical systems, uncertainty is the rule rather than the exception. Decisions depend on partial, ambiguous, or noisy evidence; data are distributed or privacy-sensitive; and stakeholders operate in cooperative–competitive environments. In this Special Issue, we bring together entropy-based methods, rough and fuzzy set formalisms [1], expert and distributed learning systems [2], and game-theoretic models to develop interpretable, robust, and privacy-aware decision support spanning areas of medicine [3], cybersecurity, and environmental risk [4].

The articles selected for this Special Issue reflect a noticeable shift in current research: from isolated modelling techniques to integrated uncertainty pipelines capable of combining robustness, interpretability, privacy awareness, and mathematical rigour. A clear example of this integration is found in the paper on interval-valued entropy measures for interval-valued fuzzy sets, where uncertainty is treated not as a nuisance but as an explicit, structured signal [5]. By embedding these interval entropies into a federated learning framework, the authors demonstrate that medical risk prediction can remain privacy-preserving while achieving high sensitivity even under heterogeneous, non-IID data distributions. The study illustrates how epistemic uncertainty, often flattened into scalar indicators, can become a valuable and interpretable component of diagnostic reasoning. A similar orientation toward uncertainty as a guiding principle appears in the paper [6], focused on entropy-based mimic-defence scheduling. Here, entropy serves not only as a statistical descriptor but also as a strategic tool. The REWS algorithm developed by the authors is grounded in incomplete-information game theory and addresses the realistic scenario of memory-based attackers operating under tight resource constraints. The paper [7] on urban flood resilience builds on this thread by showing how heterogeneous data modalities—numerical, interval, and linguistic—can be naturally merged into a unified evaluation framework. By combining SW-GAHP weighting with cloud-model fusion, the authors capture interpersonal and intrapersonal consistency while preserving the inherent semantic uncertainty in expert assessments. Uncertainty also lies at the heart of the hybrid weighting scheme [8], which responds to a long-standing problem in multi-criteria decision making: entropy weights are sensitive to outliers, while purely statistical dispersion measures may disregard meaningful variability. By integrating IQR-based robustness and entropy-based information, the authors create a weighting model that adapts smoothly to the level of contamination within the data. Recent investigations have further illuminated the critical role of normalization choices in entropy-based multi-criteria decision analysis, particularly their impact on ranking stability amid data variability. Such data-driven perspectives underscore the need for preprocessing strategies that enhance empirical robustness, ensuring that entropy weights remain reliable across diverse and uncertain decision scenarios. A more fundamental analysis of uncertainty appears in [9], where a method for cloud-model similarity is presented. This enhancement corrects systematic similarity overestimation, improves concept discrimination, and yields more reliable performance in multi-expert decision settings and time-series classification. Its conceptual cornerstone is straightforward yet powerful: uncertainty should be decomposed, not collapsed. The contribution on coalition-based decision trees [10] provides a compelling perspective on distributed decision making, where conflicts between independently maintained data sources are inevitable. By combining Pawlak’s conflict analysis, coalition formation, decision tree induction, and decision-template fusion, the authors develop a transparent and powerful methodology for reducing global decision entropy in multi-source environments.

In conclusion, this collection of papers serves as a coherent response to the growing need for decision-support systems that acknowledge and exploit uncertainty rather than suppress it. Across various domains—including medical diagnostics, cyber defence, environmental assessment, statistical weighting, cloud-model reasoning, and distributed classification—the authors show that entropy and related constructs provide not only mathematical elegance but also operational value.

## 2. Key Research Gaps

Contemporary research on uncertainty-aware decision making often confronts limitations that stem from oversimplified representations of uncertainty, insufficient robustness to data imperfections, or a lack of mechanisms that reconcile heterogeneous or conflicting information. A recurring challenge is the difficulty of capturing epistemic uncertainty in a form that preserves its structure rather than collapsing it into single-point indicators. Approaches based on interval entropies demonstrate how uncertainty can be quantified without losing the nuance of incomplete or imprecise evidence, particularly in settings where privacy constraints and fragmented information make classical aggregation impractical.

A further obstacle for complex decision problems arises from heterogeneous data sources, inconsistent expert judgments, and mixed information formats. Real-world systems rarely rely on a single data modality, and traditional multi-criteria methods have struggled to combine numerical precision, linguistic descriptions, and interval uncertainty in a principled way [11,12]. Methodologies that fuse weighting schemes with cloud-model reasoning offer a pathway toward unified treatment of multi-modal evidence, ensuring that ambiguity, vagueness, and variability are not treated as noise but as meaningful components of the decision process.

Equally important is the tendency for information-theoretic weights to become unstable in the presence of noise or outliers [13]. Pure entropy weighting is highly sensitive to numerical irregularities, while dispersion-based weights such as those derived from variability measures often disregard valuable informational structure. Hybrid approaches that explicitly balance robustness and information sensitivity create a more adaptive weighting mechanism—one capable of responding to contamination levels and shifting data distributions in a controlled and interpretable way.

Distributed decision-making environments often rely on implicit or opaque aggregation mechanisms, making it difficult to trace how conflicting or incomplete data influence the final outcome [14]. Structured approaches that combine conflict analysis, coalition formation, and interpretable model fusion address this gap by offering transparent mechanisms for understanding how individual data sources contribute to collective decisions, especially when these sources disagree or vary in reliability.

Closing these gaps requires viewing uncertainty not as a residual quantity to be suppressed, but as a foundational principle that shapes how information is represented, combined, and interpreted. By embracing this richer and more structural understanding of uncertainty, future methods can advance toward decision processes that are not only more accurate, but also more transparent, resilient, and attuned to the complexity of real-world environments.

## 3. Future Research Directions

Looking ahead, several broad research trajectories appear increasingly important for the evolution of uncertainty-aware decision making—directions that extend well beyond the methods and case studies explored so far, and that will likely shape the next decade of developments in this field. A first direction involves the construction of multi-layered uncertainty architectures capable of operating seamlessly across scales, modalities, and degrees of abstraction [15]. Future systems will need to integrate probabilistic inference, fuzzy semantics, interval representations, causal reasoning, and learning-based uncertainty estimates into an integrated conceptual and computational framework.

A second major opportunity lies in developing autonomous systems that can negotiate uncertainty, not just model it. As AI agents increasingly interact with one another—and with human decision makers—mechanisms for uncertainty-aware negotiation, coordination, and conflict resolution will become indispensable [16]. Research is needed on protocols in which agents exchange uncertainty-qualified information, justify their recommendations, and collaboratively decide when to defer, escalate, or abstain. These mechanisms must be flexible enough to operate in mixed human–machine teams and robust enough to withstand adversarial manipulation.

Equally significant is the emerging need to rethink data quality and trustworthiness in environments where data may be incomplete, strategic, corrupted, or intentionally deceptive. Traditional assumptions of stationarity and benign noise no longer hold. Future work will need to integrate provenance tracking, explainable uncertainty diagnostics, trust scores, and mechanisms for detecting epistemic anomalies. This includes designing learning systems capable of identifying when uncertainty arises from insufficient evidence, when it signals concept drift, and when it reflects adversarial activity [17].

In parallel, the rise of complex, high-dimensional data streams—sensor networks, multimodal monitoring, autonomous vehicles, remote healthcare—demands advances in real-time uncertainty quantification [18,19]. Future systems must be capable of updating uncertainty assessments not just at inference time but continuously, reflecting evolving contexts, shifting environmental conditions, and newly acquired evidence. Lightweight yet expressive representations of uncertainty will be crucial for enabling such dynamic adaptation under computational constraints.

Taken together, these research directions suggest a future in which uncertainty is not something to be avoided but something to be used. In such a future, intelligent systems will recognize uncertainty as a source of information and context rather than a weakness. This shift can lead to decision-making approaches that are not only more advanced technically, but also better suited to the complexity, unpredictability, and interconnected nature of the real environments in which they operate.

## References

[1] Singh J., Ray S.S. (2025). Integrating fuzzy rough set-based entropies for identifying drug-resistant miRNAs in cancer. J. Comput. Sci..

[2] Kalakoti R., Nõmm S., Bahsi H. (2025). Federated Learning of Explainable AI (FEDXAI) for deep learning-based intrusion detection in IoT networks. Comput. Netw..

[3] Reis M.I., Gonçalves J.N., Cortez P., Carvalho M.S., Fernandes J.M. (2025). A context-aware decision support system for selecting explainable artificial intelligence methods in business organizations. Comput. Ind..

[4] Islam S., Basheer N., Papastergiou S., Ciampi M., Silvestri S. (2025). Intelligent dynamic cybersecurity risk management framework with explainability and interpretability of AI models for enhancing security and resilience of digital infrastructure. J. Reliab. Intell. Environ..

[5] Pękala B., Kosior D., Rząsa W., Garwol K., Czuma J. (2025). Unique Method for Prognosis of Risk of Depressive Episodes Using Novel Measures to Model Uncertainty Under Data Privacy. Entropy.

[6] Wu X., Wang M., Cai Y., Chang X., Liu Y. (2025). Improving the CRCC-DHR Reliability: An Entropy-Based Mimic-Defense-Resource Scheduling Algorithm. Entropy.

[7] He X., Hu Y., Yang X., Wang S., Wang Y. (2024). Urban Flood Resilience Evaluation Based on Heterogeneous Data and Group Decision-Making. Entropy.

[8] Erbey A., Fidan Ü., Gündüz C. (2025). A robust hybrid weighting scheme based on IQRBOW and Entropy for MCDM: Stability and advantage criteria in the VIKOR framework. Entropy.

[9] Pu J., Liu Z. (2025). HECM-Plus: Hyper-Entropy Enhanced Cloud Models for Uncertainty-Aware Design Evaluation in Multi-Expert Decision Systems. Entropy.

[10] Kusztal K., Przybyła-Kasperek M. (2025). Distributed Data Classification with Coalition-Based Decision Trees and Decision Template Fusion. Entropy.

[11] Xiao Y., Ma X., Zhan J. (2026). Group decision-making in heterogeneous multi-scale information fusion: Integrating overconfident and non-cooperative behaviors. Inf. Fusion.

[12] Libório M.P., Ekel P., D’Angelo M.F.S.V., Martínez L., Pedrycz W. (2025). Dealing with heterogeneous information in multi-criteria group decision-making problems: A comprehensive design framework. IEEE Access.

[13] Alastal H., Sharaf A., Mahmoud S., Alsaidi O., Bahroun Z. (2025). Integrating Multiple Criteria Decision-Making Techniques in Sustainable Supplier Selection: A Comprehensive Review. Decis. Mak. Appl. Manag. Eng..

[14] Tong S., Sun B., Zhang L., Chu X. (2024). An approach of multi-criteria group decision making with incomplete information based on formal concept analysis and rough set. Expert Syst. Appl..

[15] Krishankumar R., Ravichandran K.S., Gandomi A.H., Kar S. (2021). Interval-valued probabilistic hesitant fuzzy set-based framework for group decision-making with unknown weight information. Neural Comput. Appl..

[16] Rowe F., Jeanneret Medina M., Journé B., Coëtard E., Myers M. (2024). Understanding responsibility under uncertainty: A critical and scoping review of autonomous driving systems. J. Inf. Technol..

[17] Korycki Ł., Krawczyk B. (2023). Adversarial concept drift detection under poisoning attacks for robust data stream mining. Mach. Learn..

[18] Chen L., Wang J., Mortlock T., Khargonekar P., Al Faruque M.A. (2025). Hyperdimensional uncertainty quantification for multimodal uncertainty fusion in autonomous vehicles perception. Proceedings of the 2025 IEEE/CVF Conference on Computer Vision and Pattern Recognition (CVPR).

[19] Yang K., Tang X., Li J., Wang H., Zhong G., Chen J., Cao D. (2023). Uncertainties in onboard algorithms for autonomous vehicles: Challenges, mitigation, and perspectives. IEEE Trans. Intell. Transp. Syst..

